# Expression Dynamics of the *O*-Glycosylated Proteins Recognized by *Amaranthus leucocarpus* Lectin in T Lymphocytes and Its Relationship With Moesin as an Alternative Mechanism of Cell Activation

**DOI:** 10.3389/fimmu.2021.788880

**Published:** 2021-11-30

**Authors:** Wilton Gómez-Henao, Rafael Saavedra, Francisco Raúl Chávez-Sánchez, Ricardo Lascurain, Edgar Zenteno, Eda Patricia Tenorio

**Affiliations:** ^1^ Departamento de Bioquímica, Facultad de Medicina, Universidad Nacional Autónoma de México, Mexico City, Mexico; ^2^ Departamento de Inmunología, Instituto de Investigaciones Biomédicas, Universidad Nacional Autónoma de México, Mexico City, Mexico

**Keywords:** *O*-glycosylation, *Amaranthus leucocarpus* lectin, moesin, T lymphocytes, costimulatory molecules

## Abstract

T lymphocyte activation begins with antigen/MHC recognition by the TCR/CD3 complex followed by a costimulatory signal provided by CD28. The search for novel costimulatory molecules has been extensive due to their potential use as immunotherapeutic targets. Although some molecules have been identified, they are unable to provide sustainable signaling to allow for proper T cell activation and proliferation. It has been shown that the *Amaranthus leucocarpus* lectin (ALL) can be used as an *in vitro* costimulator of CD4^+^ lymphocytes in the presence of anti-CD3 mAb; this lectin specifically recognizes *O*-glycans of the Galβ1-3GalNAc-O-Ser/Thr type, including a 70-kDa moesin-like protein that has been suggested as the costimulatory molecule. However, the identity of this molecule has not been confirmed and such costimulation has not been analyzed in CD8^+^ lymphocytes. We show herein that the expression kinetics of the glycoproteins recognized by ALL (gpALL) is different in CD4^+^ and CD8^+^ T cells, unlike moesin expression. Results from IP experiments demonstrate that the previously described 70-kDa moesin-like protein is an *O*-glycosylated form of moesin (*O*-moesin) and that *in vitro* stimulation with anti-CD3 and anti-moesin mAb induces expression of the activation molecules CD69 and CD25, proliferation and IL-2 production as efficiently as cells costimulated with ALL or anti-CD28. Overall, our results demonstrate that *O*-moesin is expressed in CD4^+^ and CD8^+^ T lymphocytes and that moesin provides a new costimulatory activation signal in both T cell subsets.

## Introduction

T cell activation is a complex process dependent on three sequential signals, the first comprises antigen/MHC recognition by the TCR/CD3 complex; the second involves the generation of a costimulatory signal induced by CD28 after CD80/CD86 recognition in antigen presenting cells (APC); and the third consists of the effect of cytokines that maintain cell survival, expansion, and polarization like IL-2, IFN-γ and IL-4 (1–3). This process begins with the immunological synapse formation, the contact interface between the T cell and the APC, where diverse proteins converge to promote stimulation, adhesion, communication, and signaling, many of which are highly dependent on the glycosidic portion of glycoproteins (4–6). A proper activation is highly dependent on costimulation since this second signal enhances the one induced by antigen recognition (7–9) and CD28 remains as the canonical costimulatory molecule in T cells (10). For example, although LFA-1 and CD2 have been proposed as alternative costimulatory molecules, the stimulus induced through these molecules is not strong enough to endure activation and allow adequate T cell effector functions. Other molecules like ICOS and OX40 appear once cell activation is complete and their costimulation provides survival and maintenance signals (11–14).

Earlier reports from our group showed that mouse and human CD4^+^ T cells stimulated *in vitro* with anti-CD3 and the *Amaranthus leucocarpus* lectin (ALL) activate and proliferate similarly to anti-CD3/CD28 activated cells (15, 16), suggesting that other molecules can generate initial costimulatory signals and, potentially, the existence of an alternate T cell co-activation mechanism. ALL identifies *O*-glycoproteins with a Galβ1-3GalNAc-O-Ser/Thr sequence, even in the presence of sialic acid residues in their structure (17). In mouse thymus, it recognizes CD4^+^ T cells in the medulla and the cortico-medullary junction, suggesting that the glycoproteins identified by ALL (gpALL) are expressed until T cells mature (18, 19).

Additionally, one of the molecules recognized by ALL in circulating mouse CD4^+^ T cells is a 70-kDa membrane *O*-glycoprotein that belongs to the ERM family (ezrin, radixin, and moesin) with high sequence homology to moesin (20). Moesin is mainly expressed in endothelial cells, platelets, T lymphocytes and to a lesser extent in some types of epithelial cells, where ezrin predominates (21, 22). It has been classically described as a non-glycosylated cytosolic protein involved in cell polarity maintenance and the interaction between membrane proteins with the cytoskeleton (23, 24); however, a few studies report its presence on the cell surface (25, 26). Moesin is a 67.8-kDa protein, composed of 577 amino acids, and its structure is comprised of a FERM or amino-terminal domain, a central or alpha-helical domain, and a moesin or carboxy-terminal domain (27); its cytosolic activity is regulated by phosphorylation of the conserved amino acid Thr^558^, which induces the protein to adopt an active conformation (28, 29). This protein is determinant for T cell homeostasis and function since moesin deficient mice show a reduced number of T cells in the periphery and lymph nodes (30, 31), and other studies have shown that suppressing moesin expression with siRNA hampers IL-2 production by T cells after *in vitro* stimulation (32).

Expanding our knowledge of costimulatory molecules in T cells is of great interest because they have become crucial targets for the treatment of different illnesses like cancer and autoimmune diseases (33, 34), turning any new molecule or mechanism involved in T cell activation into a potential clinical target. Therefore, in this study, we aimed to determine gpALL and moesin expression dynamics in CD4^+^ and CD8^+^ T cells after *in vitro* activation and if the 70-kDa glycoprotein detected by ALL is an *O*-glycosylated form of moesin. Finally, we evaluated if *in vitro* stimulation with anti-CD3 and anti-moesin could induce activation and proliferation of CD4^+^ and CD8^+^ T lymphocytes.

## Materials and Methods

### Mice

Six to eight-week-old male BALB/c mice weighing 28-30 g were used for all experiments. Animals were bred and maintained at our animal house following institutional guidelines and used according to the protocol approved by the institutional ethics and research committees.

### Antibodies, Lectins, and Dyes

The following fluorochrome-conjugated mAbs were used: anti-CD4-APC (RM4-5, Tonbo Biosciences), -CD4-FITC (GK1.5), -CD8-PE (53-6.7) -CD8-PerCP-Vio 700 (53-6.7), -CD25-PE (REA568), -CD69-PE-Vio770 (H1.2F3) from Miltenyi Biotec and –moesin-PE (EP1863Y, Abcam). Galβ-1,3GalNAcα1-O-Ser/Thr type *O*-glycoproteins were detected using ALL, an in-house purified lectin obtained as previously described (35). ALL was biotinylated with the EZ-Link Sulfo-NHS-Biotin kit (Thermo Fisher) following the manufacturer’s instructions using a 1:2 lectin:biotin ratio. Biotinylated ALL was detected with Streptavidin Brilliant Violet 421 (Strp BV 421, BioLegend). CFSE (Thermo Fisher) was used for cell proliferation analysis and Ghost Dye Red 780 (Tonbo Biosciences) or 7-AAD (Thermo Fisher) for dead cell exclusion.

### Cell Staining and Flow Cytometry

Cell surface molecules analysis was performed incubating 10^5^ cells with ALL followed by Strp BV 421, washed, and stained with the indicated antibody cocktail. All incubations were conducted in 100 µl of wash buffer (DPBS supplemented with 1% FCS) for 30 min (4°C, in the dark). For viability determination, cells were incubated with 0.5 µg/ml of Ghost Dye Red 780 (20 min, RT) or 1 µg/ml of 7AAD (20 min, 4°C) in DPBS (Thermo Fisher). For moesin detection, cells were fixed with 4% paraformaldehyde in DPBS for 40 min before incubation with anti-moesin as described above. Stained cells were suspended in 200 µl of DPBS, 100 µl of the cell suspension was acquired using a MACSQuant Analyzer (Miltenyi Biotec) and analyzed with FlowJo software V.10.6.2 (Beckton Dickinson).

### Proliferation Assay

Splenocytes were obtained by perfusion with DPBS and red cells were removed using hypotonic NH_4_Cl lysing buffer. Cells were stained with CFSE (Thermo Fisher) as previously reported (36) and T lymphocytes were purified by negative selection using the Pan T cell Isolation Kit II (Miltenyi Biotec). Cells (2.5 × 10^5^ cells/ml) were incubated in complete RPMI medium (RPMI 1640 supplemented with 2 mM L-glutamine, 10 mM non-essential amino acids, 1 mM sodium pyruvate, 25 mM HEPES, 50 mM 2-β mercaptoethanol, and 50 U/ml penicillin-streptomycin from GIBCO and 10% FCS (ByProducts) in 96-well plates (Sarstedt) at 37°C, 5% CO_2_. Cells were stimulated with 3 µg/ml soluble anti-CD3 (145-2C11, in house purified) and 3 µg/ml soluble anti-CD28 (37.51; Tonbo Biosciences), 5 µg/ml ALL, and/or 5 µg/ml anti-moesin (EP1863Y, Abcam). Seventy-two hours later cells were harvested, washed (DPBS, 1% FCS), and stained with the corresponding mAb combination.

### IL-2 Determination

Purified T cells stimulated as described above (2.5 × 10^5^) cells/ml were incubated at 37°C, 5% CO_2_ in RPMI complete medium in 24-well plates, and supernatants were collected at 48 h post-activation. IL-2 was quantified using the Mouse IL-2 ELISA Matched Antibody Pair Kit, following the manufacturer’s indications (Tonbo Biosciences); the detection limit was 2 pg/ml.

### Cell Sorting

Freshly obtained splenocytes (1 × 10^8^) or stimulated with 3 μg/ml anti-CD3 and 0.3 μg/ml CD28 for 48h, were labeled with anti-CD4, anti-CD8, and 7-AAD. Viable CD4^+^ and CD8^+^ gates were sorted, collected in FCS, washed several times in DPBS, and lysed immediately to obtain protein extracts; the purity of each population was ≥95%, the gating strategy and post-sort purity are shown in **Supplementary Figure 1**. Cells were sorted using a FACSAria (Beckton Dickinson) at LabNalCit, UNAM, Mexico.

### Protein Extraction

Purified CD4^+^ or CD8^+^ T lymphocytes (1 × 10^7^) were resuspended in 200 µL RIPA buffer supplemented with 2 mM Na_3_O_4_V, a phosphatase inhibitor, and EDTA-free Protease Inhibitor Cocktail 1x (Merck). The suspension was sonicated (30 s), incubated (30 min), and centrifuged for 20 min at 10,000 rpm. The supernatant was transferred to an Amicon Ultra-0.5 Centrifugal Filter Unit 10-kDa tube (Merck) for buffer exchange, centrifuging three times at 10,000 rpm for 15 min with 300 µl DPBS supplemented with phosphatase and protease inhibitors. A final volume of 200 µl of concentrated protein was obtained and concentration was determined using the PierceTM BCA Protein Assay Kit (Thermo Fisher). The T cell protein extract was then used for immunoprecipitation assays, western or lectin blots.

### Immunoprecipitation and Pull Down

For moesin immunoprecipitation experiments, 5 µl of unconjugated anti-moesin mAb (EP1863Y, Abcam) was mixed with 200 µl of the T cell protein extract (200 µg/ml) and incubated overnight at 4°C; then, 50 µl of µMACS Protein A Microbeads (Miltenyi Biotec) were added (2 h, 4°C). The immunoprecipitate’s washing, elution, and separation were performed according to the manufacturer’s recommendations. For gpALL pull-down, 200 µl of the T cell protein extract (200 µg/ml) was mixed with 30 µL of biotinylated ALL (2.2 mg/ml) and incubated at 4°C overnight; then, 100 µl of Streptavidin MicroBeads (Miltenyi Biotec) were added and further incubated for 2 h, at 4°C; the precipitate’s elution, washing, and separation were performed with 500 mM GalNAc following manufacturer’s instructions. The precipitates were used in later experiments.

### SDS-PAGE

Twenty µg of protein per well were loaded with 4x Laemmli + buffer 10% β-mercaptoethanol (Bio-Rad) at a 1:4 ratio and, later, the proteins were subjected to 10% SDS-PAGE (Invitrogen™) using Precision Plus Dual Color molecular weight standards (MW, Bio-Rad) as reference. Afterward, the gel was transferred to a polyvinyl difluoride (PVDF) Immobilon-P membrane (Millipore Corp). Ponceau S (Sigma-Aldrich) was used as a loading control in the experiments during the identification experiments of the glycans recognized by ALL.

### Lectin and Western Blot

For lectin-blot, the PVDF membrane was blocked with T-PBS (PBS + 0.1% v/v Tween), 3% BSA for 1 h at room temperature, incubated with ALL 1.1 µg/ml in PBS overnight (4°C) and washed 3 times with T-PBS followed by incubation with Streptavidin HRP (Strp-HRP, 1:7000; Vector Laboratories) in T-PBS, 5% Blotto-non-fat dry milk (Santa Cruz Biotechnology) for 1 h at room temperature and a final wash with T-PBS, 0.2% Triton X-100. Western-blot membranes were blocked for 1 h at room temperature using T-TBS (Tris-buffered saline + 0.1% Tween), 3% BSA, followed by incubation with anti-moesin HRP (EP1863Y, 1:5000; Abcam) diluted in TBS overnight at 4°C and washed with T-TBS, 0.2% Triton X-100. Both membranes were developed using Immobilon Western Chemilum HRP Substrate (Merck) on autoradiographic Kodak Biomax films (Sigma-Aldrich), and data were analyzed using Lab 6.0.1 software (Bio-Rad).

### Statistical Analysis

Statistical differences between groups were determined using one-way ANOVA with Bonferroni’s multiple comparison tests using PRISM software V9.2 (GraphPad).

## Results

**Figure 1:**
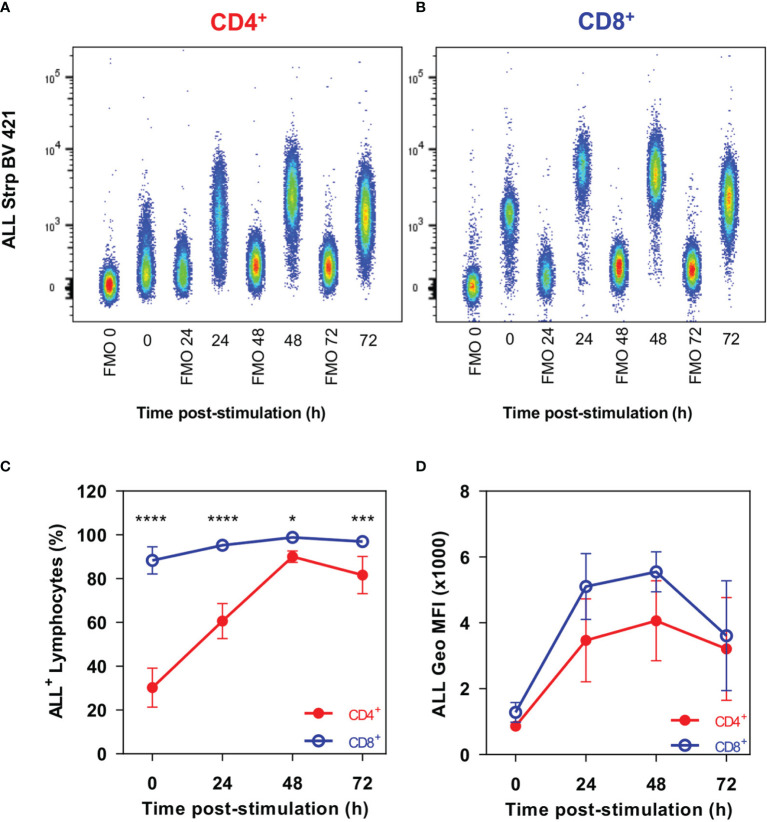
CD4^+^ and CD8^+^ T cells show different gpALL expression patterns. Splenocytes were stimulated with anti-CD3 and anti-CD28 mAbs; cells were harvested at the indicated time points and stained with anti-CD4, anti-CD8, ALL, Ghost Dye Red 780, and immediately analyzed in the flow cytometer. Lymphocytes were defined by FCS and SSC characteristics, after singlets selection, dead cells were excluded and gpALL were analyzed within CD4^+^CD8^-^ and CD4^-^CD8^+^ sub gates. Representative analysis of gpALL expression kinetics in **(A)** CD4^+^ and **(B)** CD8^+^ T cells; **(C)** percentages of CD4^+^ALL^+^ and CD8^+^ALL^+^ cells and **(D)** ALL binding in each cell subset after activation. Mean and SD from 2 independent experiments with 3 mice per group are depicted in C and D; data were analyzed using one-way ANOVA followed by Bonferroni’s multiple comparison test between CD4^+^ (Red full circles) and CD8^+^ (Blue open circles) at each time point. *p < 0.05, ***p < 0.0005, ****p < 0.0001.

We first performed a comparative analysis of gpALL expression kinetics by flow cytometry in CD4^+^ T and CD8^+^ T lymphocytes stimulated for 24 h, 48 h, and 72 h with anti-CD3 and anti-CD28 mAbs (**Figure 1**). Results show that 30% of unstimulated CD4^+^ T lymphocytes are ALL^+^, this percentage doubles 24 h post-activation and reaches its maximum after 48 h, when 90% of CD4^+^ cells are recognized by ALL (**Figures 1A, C**). In contrast, 88% of not activated CD8^+^ lymphocytes already express gpALL and peak positivity occurs at 48h post-activation where 98.8% of cells are ALL^+^ (**Figures 1B, C**); in both cases, a decrease of ALL binding is observed at 72 h (**Figures 1A, B, D**). Despite the difference in cell percentage positivity, gpALL expression was equivalent in both unstimulated T cell subsets, and in both cases, peak expression was reached at 48 h post-activation; gpALL expression increased 4.7 times in CD4^+^ cells (**Figures 1A, D**) and 4.3 times in CD8^+^ cells (**Figures 1B, D**). Although gpALL expression increases in both cell subsets as a consequence of activation, almost 90% of unstimulated CD8^+^ cells are ALL^+^; showing that gpALL is constitutively expressed in this cell subset but inducible in CD4^+^ cells (**Figure 1**).

**Figure 2:**
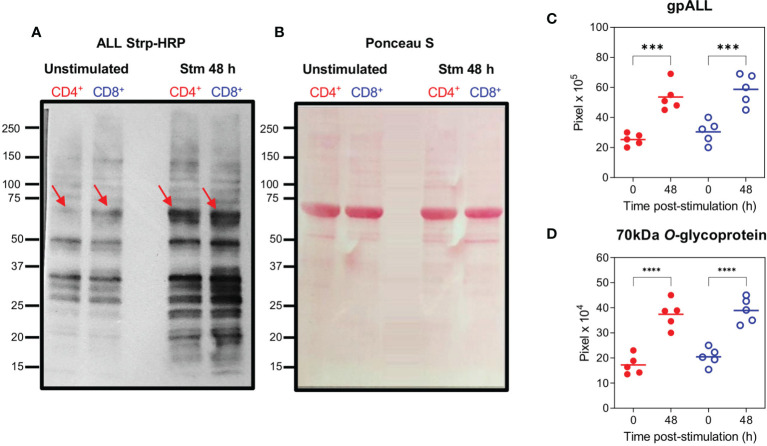
*O*-glycoproteins detected by ALL are increased on CD4^+^ and CD8^+^ T cells after activation. Splenocytes were stimulated with anti-CD3 and anti-CD28 mAbs for 48 h, after harvesting, CD4^+^ and CD8^+^ T cells were separated by cell sorting and protein extracts were obtained. Samples were analyzed by lectin blot using ALL Strp-HRP **(A)**, *O*-glycoproteins from unstimulated (Lanes 1 and 2) and stimulated (Lanes 3 and 4) from CD4^+^ (Lanes 1 and 3) and CD8^+^ (Lanes 2 and 4) cells are shown; the red arrow indicates the previously reported 70-kDa *O*-glycoprotein recognized by ALL; the protein profile transferred to a PVDF membrane and stained with Ponceau S is included as a loading control **(B)**. Quantification of the accumulative intensity from the total number of bands per lane **(C)** and the 70-kDa band recognized by ALL **(D)** from 5 independent experiments pooling the cells from 2 animals per group. Data were analyzed using ANOVA followed by Bonferroni’s multiple comparison test between unstimulated and stimulated (CD4^+^ (Red full circles) and CD8^+^ (Blue open circles); ***p < 0.001, ****p < 0.0001.

To further analyze these differences, we evaluated the *O*-glycoprotein profile recognized by ALL in unstimulated and 48 h stimulated cells with anti-CD3/CD28 by lectin blot. These results (**Figures 2A, C**) revealed that in both CD4^+^ and CD8^+^ T cells the number of glycoproteins and the intensity of the bands recognized by ALL increased after 48 h of activation compared to the gpALL expressed in unstimulated cells (**Figures 2A–C**). ALL recognized *O*-glycoproteins with molecular weights that vary between 20 and 150-kDa in both stimulated and unstimulated cells; after stimulation, over twice the bands were detected in CD4^+^ cells (Unstimulated 7 vs Stimulated 15), while 5 additional bands were detected in CD8^+^ cells (unstimulated 11 vs Stimulated 16) (**Figure 2A**). Quantitative analysis of the glycoproteins recognized by ALL (**Figure 2C**), showed that the mean gpALL expression level increased 1.1 times after activation in CD4^+^ T cells and 0.8 times in CD8^+^ T cells compared to unstimulated samples. Within these, the 70-kDa protein (**Figure 2A**, arrow) is of particular interest for us because its expression has been previously reported in T cells from the thymus, peripheral blood, lymph nodes, and spleen (18). Herein we found an increased expression of the 70-kDa protein in CD4^+^ and CD8^+^ T cells after 48 h stimulation (1.2 and 0.9 times, respectively. **Figure 2A**, arrows, and **2D**).

**Figure 3:**
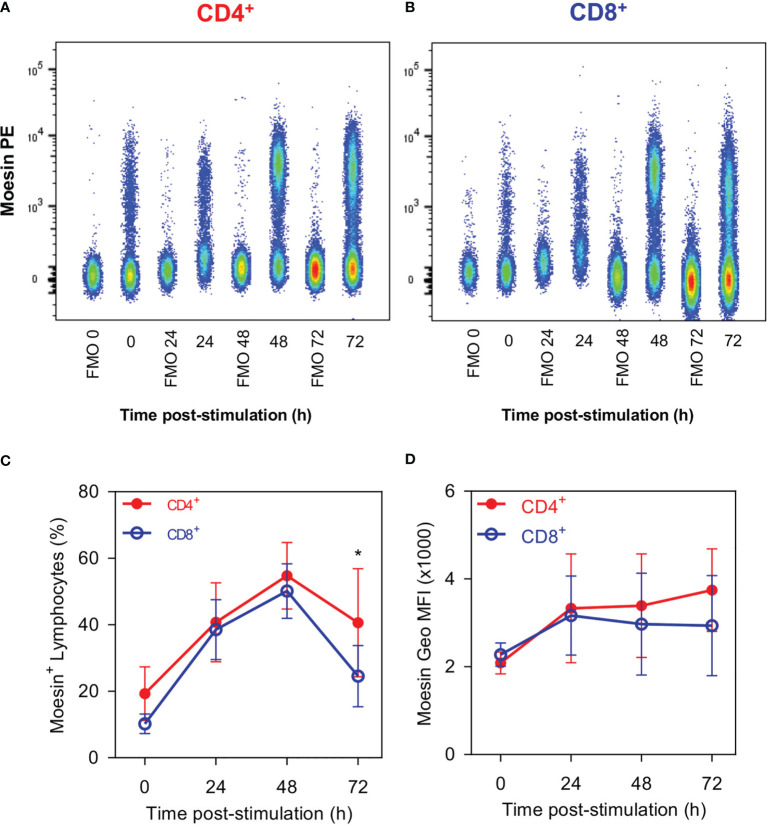
CD4^+^ and CD8^+^ Lymphocytes show similar moesin expression. Splenocytes were stimulated with anti-CD3 and anti-CD28 mAbs; cells were harvested at the indicated time points and stained with anti-CD4, anti-CD8, anti-moesin, Ghost Dye Red 780, and immediately analyzed by flow cytometry. CD4^+^ and CD8^+^ gates were defined as described in **Figure 1**. Representative analysis of moesin expression kinetics in **(A)** CD4^+^ and **(B)** CD8^+^ T cells; **(C)** percentages of CD4^+^ moesin^+^ and CD8^+^ moesin^+^ cells and **(D)** moesin expression in each cell subset after activation. Mean and SD from 2 independent experiments with 3 mice per group are shown in C and D; data were analyzed using one-way ANOVA followed by Bonferroni’s multiple comparison test between CD4^+^ (Red full circles) and CD8^+^ (Blue open circles) at each time point. *p < 0.05.

This 70-kDa protein has been identified as an ERM family member with high sequence homology to moesin (20), thus, we evaluated the membrane surface expression kinetics of moesin by flow cytometry. As can be seen in **Figures 3A–C**, some unstimulated CD4^+^ and CD8^+^ T cells were moesin^+^ (19.2 and 10.25%, respectively), these percentages increased up to 54.8% in CD4^+^ cells and 50.2% in CD8^+^ cells 48 h after stimulation. Consequently, membrane moesin expression increased slightly in both cell subsets after stimulation, up to 1.8 times in CD4^+^ T cells at 72 h and 1.4 times in CD8^+^ T at 24 h (**Figures 3A, B, D**). These results demonstrate that moesin is expressed in the cell membrane and the expression kinetics is equivalent in CD4^+^ and CD8^+^ cells after activation.

**Figure 4:**
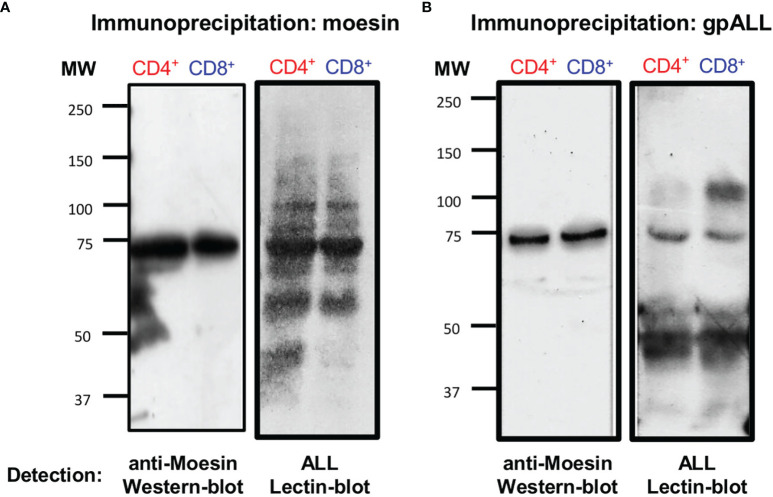
Moesin is the 70-kDa *O*-glycoprotein recognized by ALL in CD4^+^ and CD8^+^ lymphocytes. T cell protein extracts from 48 h stimulated cells were obtained as described in **Figure 2** and precipitated with **(A)** anti-moesin mAb or **(B)** ALL using the µMACS separation system. Proteins were separated by SDS-PAGE, then, western and lectin blots were developed with anti-moesin (**A** and **B**, left) and ALL (**A** and **B**, right), respectively. The experiment was performed twice with comparable results.

Arenas et al. also showed that this moesin-like protein co-localized with gpALL on the cell surface of CD4^+^ lymphocytes after activation (20). Thus, to demonstrate that an *O*-glycosylated form of moesin (*O*-moesin) is expressed in both CD4^+^ and CD8^+^ cell subsets, we performed precipitation and cross-recognition assays using an anti-moesin mAb and ALL in protein extracts from 48 h activated CD4^+^ and CD8^+^ T lymphocytes. First, we immunoprecipitated the proteins with anti-moesin, and performed a lectin-blot using ALL and a western-blot developing with anti-moesin (**Figure 4A**), results showed that ALL recognizes 5 proteins with equivalent molecular weights in both cell subsets (150, 100, 80, 70 and 60-kDa) and an additional 45-kDa protein in CD4^+^ cells only. The 70-kDa band was the most prominent in both cell subsets and corresponds to the only band observed in the anti-moesin western blot. In parallel, ALL precipitated proteins were analyzed in a western blot developed with anti-moesin, where a single 70-kDa band is revealed in both cell subsets and a lectin blot with ALL (**Figure 4B**). The latter showed the presence of 5 bands in CD4^+^ T lymphocytes and 4 bands in CD8^+^ T, including the 70-kDa O-glycoprotein. These results show that ALL recognizes an *O*-moesin expressed in CD4^+^ and CD8^+^ T lymphocytes, the detection of additional molecules in the lectin blots, suggests that these molecules could have been isolated as part of a larger molecular complex.

Up to date, no reports describe the presence of a glycosylated moesin form. Prediction of possible *O*-glycosylation sites using NetOGlyc 4.0 server (**Supplementary Figure 2**) showed that among the 577 amino acids that integrate moesin, only 3.8% of them have > 50% probability to be Ser/Thr *O*-glycosylated: 1 at the FERM domain, 13 at the alpha-helical domain, and 8 at the moesin domain. Only 6 sites have > 80% probability of being *O*-glycosylated, within this group Thr^469^ has the higher probability of modification (95%) but Thr^558^ (84%), located at the moesin domain, is particularly interesting because it has been reported as a fundamental site for the activation and inhibition of intracellular moesin (23).

Given that ALL can provide a costimulatory signal for T cells similar to the one provided by anti-CD28 in the presence of anti-CD3 *in vitro* and that we have demonstrated herein that *O*-moesin is recognized by this lectin, we aimed to determine if we could accomplish a similar activation profile after moesin costimulation. To this end, purified T lymphocytes were cultured in the presence of anti-CD3 and anti-moesin for 72 h, and different activation parameters were evaluated: CD69 and CD25 expression, cell proliferation, and IL-2 production.

**Figure 5:**
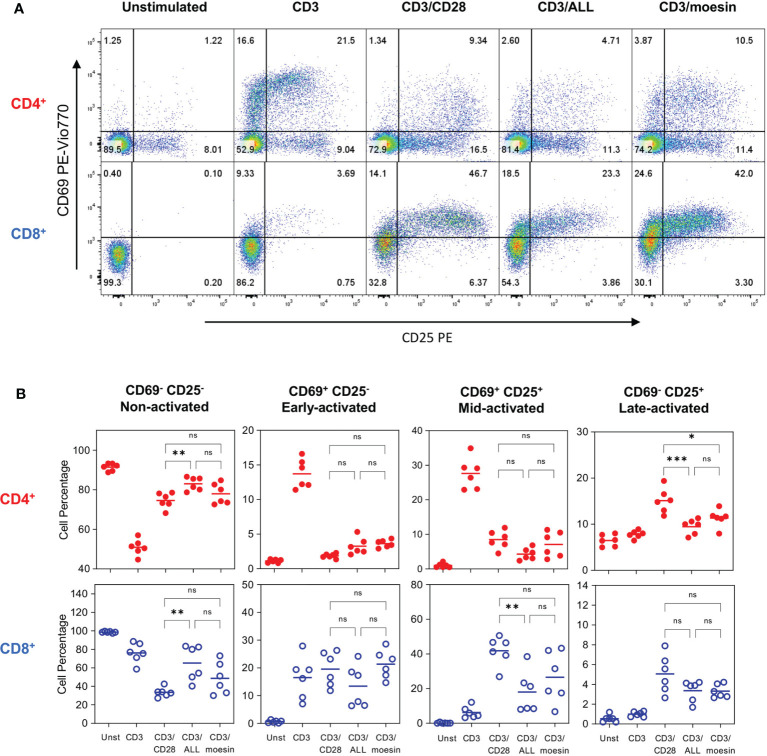
Moesin costimulation induces the expression of activation markers in T lymphocytes. Splenocytes were stained with CFSE, then, T cells were isolatedby negative selection and activated with anti-CD3 and anti-CD28 mAbs (CD3/CD28), anti-CD3 and ALL (CD3/ALL), or anti-CD3 and anti-moesin mAb (CD3/moesin) and collected after 72h. Samples were stained with anti-CD4, anti-CD8, anti-CD69, anti-CD25, Ghost Dye Red 780, and analyzed by flow cytometry. Lymphocytes were defined by FCS and SSC characteristics, after singlets selection, dead cells were excluded, and the activation markers were analyzed within the CD4+CD8- and CD4-CD8+ subsets. Representative analysis of CD69 and CD25 expression pattern in CD4+ and CD8+ T cells **(A)**, the gates indicate 4 activation stages: CD69-CD25- (Non-activated), CD69+CD25- (Early activated), CD69+CD25+ (Mid-activated) and CD69+CD25+ (Late activated). **(B)** Percentages of the cell proportions at each activation stage after stimulation with anti-CD3 and each costimulatory molecule from 2 independent experiments with 3 mice per group. Data were analyzed using one-way ANOVA followed by Bonferroni’s multiple comparison test between the indicated conditions. ns, not significant, *p < 0.05 **p < 0.001, ***p < 0.001. Additional controls performed in this experiment included cells stimulated with anti-CD28, ALL or anti-moesin only, anti-CD28/ALL, or anti-CD28/anti-moesin areshown in **Supplementary Figure 1**.

**Figure 5A** shows a representative CD69 and CD25 expression analysis in both T cell subsets 72 h after activation. Analysis of CD4^+^ cells stimulated with CD3/moesin showed that 3.87% had a very early activated phenotype (CD69^+^CD25^-^), 10.5% had a mid-activation phenotype (CD69^+^CD25^+^) and 11.4% showed a late activation phenotype (CD69^-^CD25^+^). Among CD3/Moesin activated CD8^+^ T cells 24.6% where at the earliest activation point (CD69^+^CD25^-^), 42% were mid-activated (CD69^+^CD25^+^) and 3.3% were at the latest activation stage (CD69^-^CD25^+^). In both cases, the comparison with control cells activated with CD3/ALL and CD3/CD28 showed a similar pattern to those observed in cells activated with CD3/moesin (**Figures 5A, B**); no differences were found when CD69 and CD25 expression was analyzed 6 h post activation (**Supplementary Figure 3**). These experiments demonstrate that moesin is a costimulator as effective as CD28.

**Figure 6:**
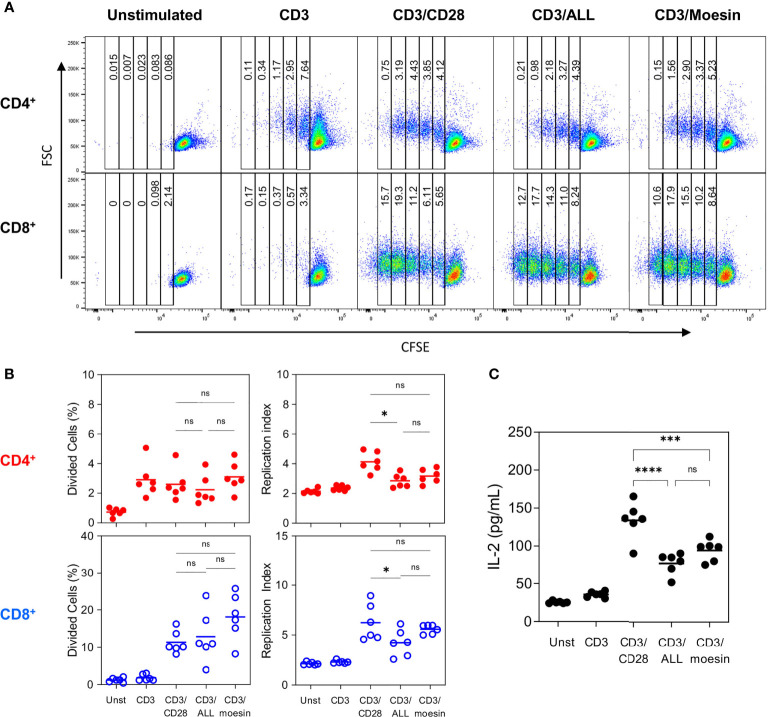
Moesin costimulation induces proliferation in CD4^+^ and CD8^+^ T lymphocytes. Samples described in **Figure 6** were further and identically analyzed within the CD4^-^ CD8^+^ and CD4^+^ CD8^+^ subsets to evaluate CFSE dilution. **(A)** Representative proliferation pattern analysis of CD4^+^ and CD8^+^ cells after activation with anti-CD3 and each costimulatory molecule. **(B)** Statistical analysis from 2 independent experiments with 3 mice per group expressed as the percentage of divided cells or as the replication index (Total Number of divided cells/The number of cells that went into division). **(C)** Negatively separated T cells were activated with anti-CD3 and anti-CD28, ALL, or anti-moesin. After 48 h, culture supernatants were collected and IL-2 concentration was determined by ELISA. Data correspond to 2 independent experiments with 3 mice per group and were analyzed using ANOVA and Bonferroni’s multiple comparison test. ns, not significant, *p < 0.05, ***p < 0.001, ****p < 0.0001. Additional controls performed in this experiment included cells stimulated with anti-CD28, ALL or anti-moesin only, anti-CD28/ALL, or anti-CD28/anti-moesin are shown in **Supplementary Figure 4**.

Cell proliferation analysis (**Figure 6**) revealed that after CD3/moesin stimulation CD4^+^ cells divided 4 times and CD8^+^ cells divided 5 times. Statistical analysis of the percentage of divided cells (**Figure 6B**) showed that 3.1% of CD4^+^ and 18.13% of CD8^+^ cells from the original population were able to divide at least once (37). A similar proliferation pattern was observed in control cells stimulated with CD3/ALL and CD3/CD28, although proliferation was slightly more efficient in the latter condition. This data demonstrates that CD3/moesin activated T cells show a classic mitogen-induced cell proliferation pattern, including the faster division rate observed in CD8^+^ cells. Finally, we evaluated IL-2 production (**Figure 6C**) in CD3/moesin stimulated T cells, where 91.33 pg/ml were detected, 3.7 times more than unstimulated cells (25.33 pg/ml), slightly but not significantly more than CD3/ALL stimulated cells (77.26 pg/ml) and less than CD3/CD28 cells (133.9 pg/ml).

These results demonstrate that costimulation through moesin is strong enough to initiate, maintain, and propagate the necessary signaling that leads to T cell activation and proliferation, outlining CD3/moesin as an alternate cell activation path. Moreover, given the similar activation and proliferation patterns observed between cells stimulated with anti-CD3 and anti-moesin or anti-CD3 and ALL, along with the results from IP experiments, it is tempting to speculate that the costimulatory signal is provided by the *O*-glycosylated form of moesin.

## Discussion

This work aimed to determine if the 70-kDa protein recognized by the *Amaranthus Leucocarpus* Lectin (ALL) in the T cell membrane is an O-glycosylated form of moesin and its potential ability to work as a CD4^+^ and CD8^+^ costimulatory molecule. Previous studies had described the presence of gpALL in mouse peripheral CD4^+^ and CD8^+^ T lymphocytes (18) however, the expression dynamics of these molecules after activation was unknown. Our results show that *in vitro* stimulation with anti-CD3/CD28 leads to an increase in ALL^+^ cells within the CD4^+^ and CD8^+^ subsets along with an increased expression of gpALL that peaks 48 h post-activation. This is consistent with the glycoprotein profile recognized through lectin blot by ALL and agrees with previous reports describing the remodeling of different glycosylation profiles after T cell activation (5, 38). Although gpALL expression increases similarly in both cell subsets after activation, it must be noted that the proportion of ALL^+^ cells is very different in freshly obtained CD4^+^ and CD8^+^ lymphocytes, which is approximately 30% and 90% respectively. This observation leads to wonder if the proportion of gpALL expressing non-activated CD4^+^ and CD8^+^ T cells is related to different activation or regulatory processes in each subset.

Among the bands recognized by ALL, the 70-kDa protein has been of particular interest. This molecule doubles its expression in CD4^+^ and CD8^+^ T cells after 48 h post-activation, which concurs with previous findings in mouse splenocytes (18), thymus (39), and macrophages (40). Further analysis of this protein showed that it is a member of the ERM family with 41% homology to an unnamed protein related to moesin (20), thus, given that its identity had not been confirmed we performed several experiments to address this issue. Although the moesin sequence contains no apparent transmembrane domain (41), it has been detected in the membrane of different cell lines (26) and the periphery of several hematopoietic cells (42). Kinetic expression analysis of cell surface moesin in CD4^+^ and CD8^+^ T cells after activation with anti-CD3/CD28 revealed that a similar percentage of moesin^+^ cells is observed within both unstimulated T cell subsets and that a similar expression dynamic is observed after activation. These observations contrast with the flow cytometric gpALL analysis, where a differential expression is observed between T cell subsets, however, we must consider that ALL recognizes several other molecules besides the 70-kDa protein and moesin expression experiments were limited by the analysis of the single protein, all of which accounts for the observed differences. While performing these experiments it caught our attention that we were only able to detect moesin by flow cytometry after cells had been fixed, it has been described that PFA alters cell surface mechanical properties due to the induction of covalent crosslinking between molecules (43), reduces cell mass density and destructs membrane integrity by dissolving some membrane lipids (44). We think that the membrane remodeling caused by PFA fixation allows the anti-moesin mAb to bind its otherwise hidden target, it is tempting to speculate that moesin is embedded in the cell membrane and that the *O*-GalNAcylation detected by ALL projects outwards. Further analysis on this structural subject will be determinant to understand moesin’s function.

The existence of the *O*-glycosylated moesin (*O*-moesin) was confirmed with IP experiments given that we were able to detect moesin after precipitation with ALL and vice versa. Interestingly, the lectin blot from the anti-moesin precipitated sample shows that besides the presence of the 70-kDa band, other proteins are detected by ALL, suggesting that moesin could have been precipitated as part of a molecular complex where other molecules share the same *O*-glycosylation. Moreover, in the lectin blot from the ALL-precipitated sample a dense 45-kDa band in CD4^+^ and CD8^+^ subsets and a 100-kDa band in CD8^+^ cells were also detected, suggesting that besides *O*-moesin, other *O*-glycosylated proteins are heavily expressed on T cells after activation. This agrees with the flow cytometry experiments where we can see a higher cell percentage positivity to ALL than moesin, particularly in the CD8^+^ subset.

Since moesin has a molecular weight of 67.8-kDa (27) and the *O*-moesin detected herein has a molecular weight of 70-kDa, we hypothesize that this difference corresponds to the glycosidic proportions of the *O*-glycoprotein, but this remains to be demonstrated by instrumental techniques in future works. Nevertheless, the bioinformatics analysis indicated that Thr^558^ is a site with a very high probability of *O*-glycosylation. This position has been described as fundamental for moesin function regulation since phosphorylation at this amino acid induces the protein to adopt an active/extended conformation where it can interact with other proteins (28, 29). Phosphorylation/*O*-glycosylation interplay has been described as a molecular switch that can regulate protein function and location (45); in fact, after T cell activation moesin is phosphorylated, and once activated it removes CD43 from the immunological synapse allowing proper activation (46). We consider that moesin Thr^588^ could be a target site for regulation through an *O*-glycosylation/phosphorylation interplay which could mediate the function and location of the protein in the cytosol or the cell membrane. Under this hypothesis, other interesting questions arise, like if *O*-moesin is synthesized *de novo* and directed to the cell membrane, or if the glycan addition to the protein occurs at the cytosol redirecting its translocation towards the cell membrane. The latter would be a rare example where the *O*-glycosylation of the T antigen type is synthesized in a place other than the endoplasmic reticulum (47); the answers to these questions are beyond the scope of this work but are proposed as thought-provoking perspectives.

Finally, we demonstrate that moesin can induce a costimulatory signal equivalent to the one provided by CD28 during *in vitro* activation with anti-CD3, considering that anti-CD3/moesin stimulated T cells were able to express activation molecules, produce IL-2 and proliferate. Although these responses were slightly lower than the ones observed after CD28 costimulation, the proliferation and activation patterns were the same between cells costimulated with ALL and anti-moesin. These results are in agreement with previous experiments reporting ALL costimulatory capacity in mouse and human T cells (15, 16) and that *O*-moesin is located in lipid rafts, cell membrane structures fundamental for the formation of the immunological synapse (20). Our observations reinforce other works describing moesin as a fundamental molecule for T cell homeostasis, maturation, and function: moesin^-/-^ mice exhibit T cells with reduced activation capacity and IL-2 production; lymphopenia as a consequence of a diminished incapacity to egress from the thymus, CD8^+^ Treg cells from these animals show decreased proliferation (30–32) and an X-linked moesin-associated immunodeficiency where diminished T cell proliferation is observed has been described in humans (48). Moesin also promotes actin polymerization during blast formation and is essential for the formation of the immunological synapse and T cell activation (32, 46, 49).

Altogether, our results demonstrate that moesin provides a, previously undescribed, costimulatory signal in CD4^+^ and CD8^+^ T cells that along with CD3 crosslinking can induce activation and proliferation; whether the signal is provided by the *O*-glycosylated form of moesin, if the activated cells can be polarized, perform cytolysis or become memory cells, and which is the natural ligand for this receptor, remain to be determined. However, our results show that T cells can be activated through a CD28 independent costimulatory pathway, which opens the door to increase our understanding of T cell biology and to investigate new potential immunomodulatory therapeutic targets.

## Data Availability Statement

The raw data supporting the conclusions of this article will be made available by the authors, without undue reservation.

## Ethics Statement

The animal study was reviewed and approved by Comité Interno para el Cuidado y Uso de Animales de Laboratorio (CICUAL), División de Investigación. Facultad de Medicina, UNAM.

## Author Contributions

WG-H, EZ, and ET designed the research study. WG-H performed the experiments. WG-H, FC-S, and ET analyzed the data. ET, RS, WG-H, and EZ wrote the article. All the authors discussed the results, reviewed the manuscript, and approved the submitted version.

## Funding

This work was supported by grants IN218719, IN204717, IN213818, IN218919, and IN200321 from PAPIIT (DGAPA, UNAM, Mexico) and by grant 251116 from CONACYT (Mexico). WGH is the recipient of a Ph.D. fellowship from CONACYT (Registry 742472). WG-H is part of Biochemical Sciences PhD Program of the Universidad Nacional Autónoma de México and of the Université Paris-Est Créteil, France.

## Conflict of Interest

The authors declare that the research was conducted in the absence of any commercial or financial relationships that could be construed as a potential conflict of interest.

## Publisher’s Note

All claims expressed in this article are solely those of the authors and do not necessarily represent those of their affiliated organizations, or those of the publisher, the editors and the reviewers. Any product that may be evaluated in this article, or claim that may be made by its manufacturer, is not guaranteed or endorsed by the publisher.
