# Supplementary material for: Expression Dynamics of the O-Glycosylated Proteins Recognized by Amaranthus leucocarpus Lectin in T Lymphocytes and Its Relationship With Moesin as an Alternative Mechanism of Cell Activation
Source: Front Immunol. 2021 Nov 30;12:788880. doi: 10.3389/fimmu.2021.788880 (PMC8669815; doi:10.3389/fimmu.2021.788880)
Supplement: Supplementary file 1 [file DataSheet_1.docx]

Supplementary Material


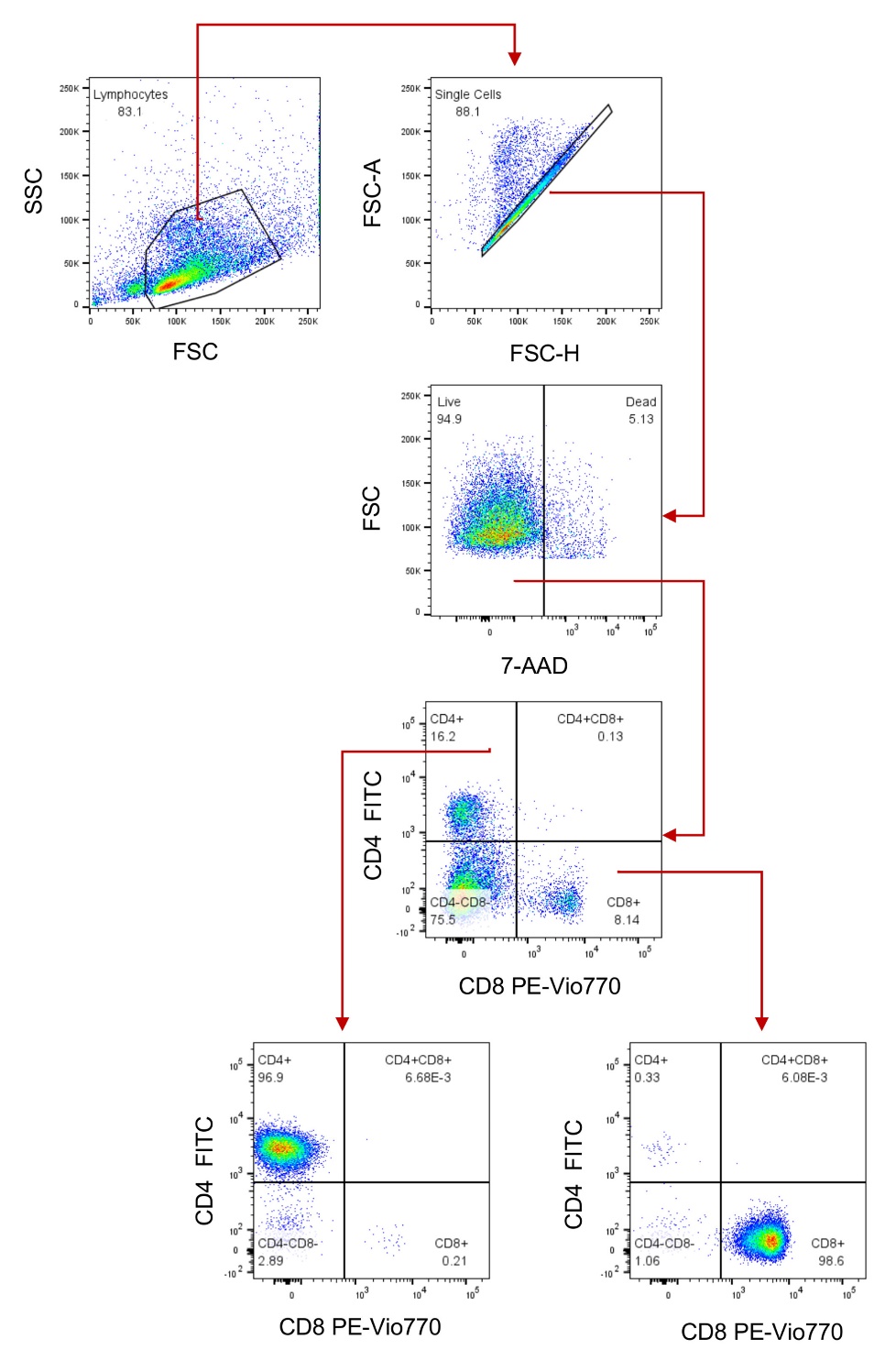


**Supplementary Figure 1**. **Cell sorting strategy and post-sort purity**. Freshly obtained splenocytes or stimulated with anti-CD3 and anti-CD28 were stained with anti-CD4, anti CD8, 7-AAD and sorted immediately. Lymphocytes were defined by FCS and SSC characteristics, after singlets selection, viable cells were gated, CD4^+^ and CD8^+^ cells were collected in FCS. Post sort purity of each population was ≥95%.


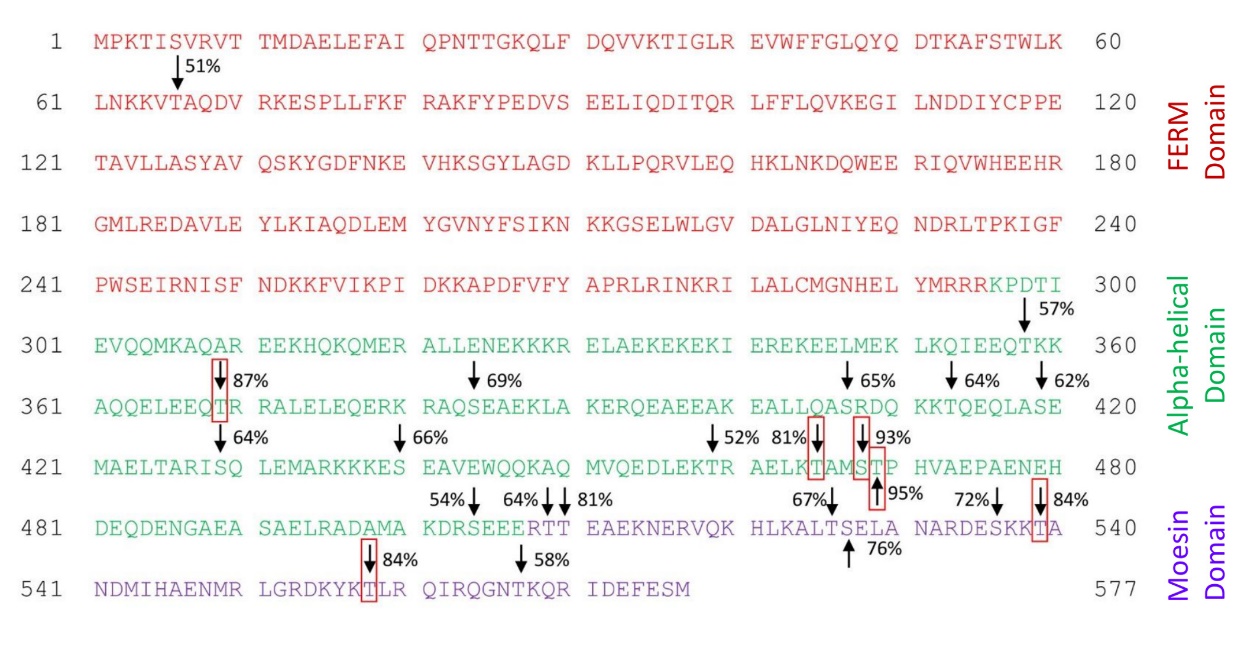


**Supplementary Figure 2**. **Moesin has several potential *O*-glycosylation sites.** The moesin amino acid sequence reported in the UniProtKB database (P26041, MOES_MOUSE) was analyzed with the NetOglyc 4.0 server to determine possible *O*-glycosylation sites. RED: FERM domain (Met^1^-Arg^95^); GREEN, alpha-helical domain (Lys^96^-Glu^506^); PURPLE, moesin domain (Arg^507^-Met^577^). Amino acids with over 50% probability of being O- glycosylated are indicated by an arrow, and those with over 80% probability are highlighted with red squares.

**
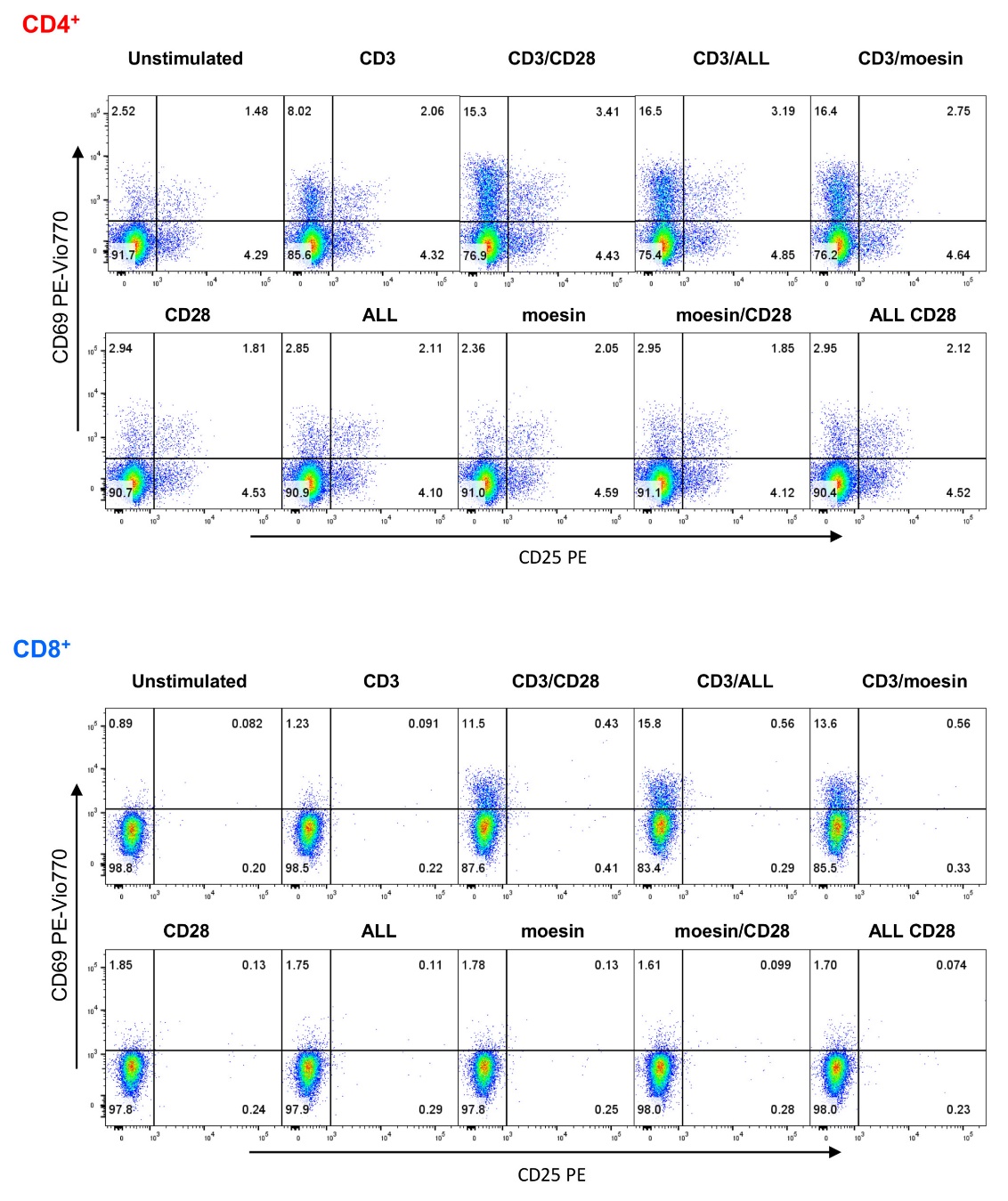
**

**Supplementary Figure 3**. **Moesin costimulation induces the expression of activation markers in T lymphocytes.** T cells were isolated by negative selection and activated with anti-CD3 and anti-CD28 mAbs (CD3/CD28), anti-CD3 and ALL (CD3/ALL), or anti-CD3 and anti-moesin mAb (CD3/moesin) and collected after 6h. Samples were stained with anti-CD4, anti-CD8, anti-CD69, anti-CD25, Ghost Dye Red 780, and analyzed by flow cytometry. Lymphocytes were defined by FCS and SSC characteristics, after singlets selection, dead cells were excluded, and the activation markers were analyzed within the CD4^+^CD8^-^ and CD4^-^CD8^+^ subsets. Representative analysis of CD69 and CD25 expression pattern in CD4^+^ and CD8^+^ T cells.


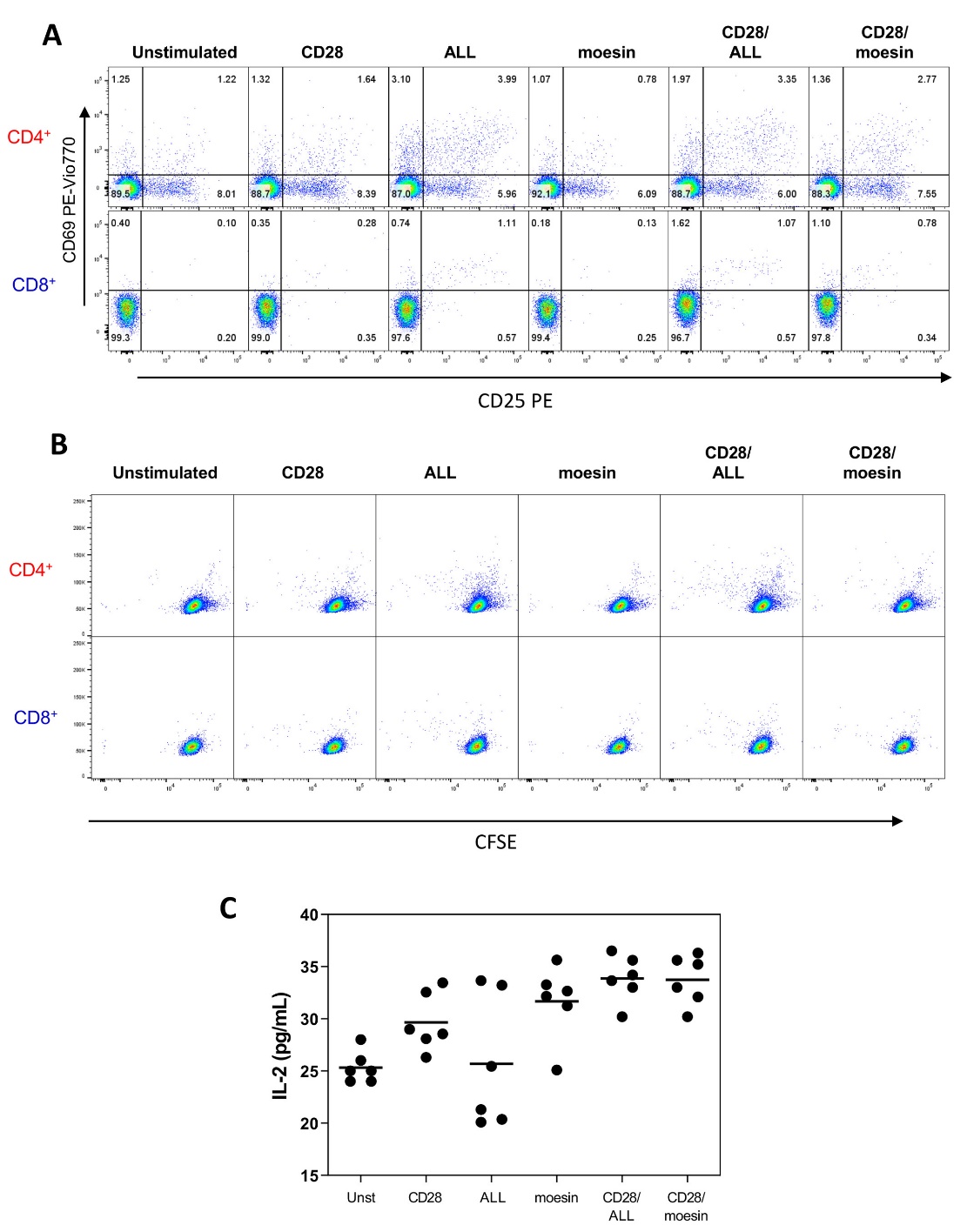


**Supplementary Figure 4**. **Additional controls included forthe activation markers expression, CFSE and IL2 secretion analysis.** Splenocytes were stained with CFSE, then, T cells were separated by negative selection and activated with, anti-CD28, ALL or anti-Moesin only, anti-CD28/ALL, or anti-CD28/anti-moesin and collected after 72h. Samples were stained with anti-CD4, anti-CD8, anti-CD69, anti-CD25, Ghost Dye Red 780, and analyzed by flow cytometry. Lymphocytes were defined by FCS and SSC characteristics, after singlets selection, dead cells were excluded. (A) Representative analysis of CD69 and CD25 expression patterns in CD4^+^ and CD8^+^ T cells is shown. (B) Representative proliferation pattern analyses of CD4^+^ and CD8^+^ cells are shown. (C) Negatively separated T cells were stimulated with, anti-CD28, ALL or anti-Moesin only, anti-CD28/ALL, or anti-CD28/anti-moesin. After 48 h, culture supernatants were collected and IL-2 concentration was determined by ELISA.
